# Dietary patterns are associated with lung function among Spanish smokers without respiratory disease

**DOI:** 10.1186/s12890-016-0326-x

**Published:** 2016-11-25

**Authors:** Mar Sorli-Aguilar, Francisco Martin-Lujan, Gemma Flores-Mateo, Victoria Arija-Val, Josep Basora-Gallisa, Rosa Sola-Alberich, for the RESET Study Group investigators, D. Borras-Vicente, F. Gomez-Santidrian, M. Grive-Isern, D. Jovani-Puig, M. T. Juncosa-Cabre, M. T. Martinez-Perez, N. Sarra-Manetas, E. Aragones-Benaiges, S. Exposito-Ribau, E. Ferrer-Sorribas, S. Folch-Pujol, M. Fores-Palacios, P. Perez-Layarra, F. Buj-Visiedo, I. Gallego-Arteaga, N. Guinjoan-Aymemi, A. Poca-Pastor, M. Vila-Molet, M. T. Canela-Armengol, J. Daniel-Diez, I. Farre-Torra, M. Garcia-Olive, M. D. Gil-Sanchez, C. Senosiain-Yerno, R. Vila-Ruiz, M. J. Andres-Pablo, J. M. Hernandez-Anguera, A. M. Lara-Pedrosa, T. Lara-Pedrosa, M. Lluis-Burgueño, M. Mengual-Miralles L. Pelleja-Pellicer, R. Rodriguez-Perez, M. Rodríguez-Miguelez, M. Sabate-Margalef, R. Subirats-Segarra, N. Allende-Muntane, T. Aviño-Llopis, M. Boira-Costa, C. Delgado-Azuara, M. Garcia-Vaque, S. Loran-Valcarcel, T. Sangra-Rodes, E. Alvarez-Soler, M. T. Basora-Gallisa, J. Blade-Creixenti, J. Breva-Aymerich, A. Caballero-Alias, A. DelPozo-Niubo, N. Martin-Vergara, A. Vinuesa-Fernandez, M. G. Aguirre-Alava, S. Crispi-Cifuentes, Y. Fernandez-Pages, A. Garcia-Uriarte, R. Solis-Narvaez, R. Caro-Garduño, C. Cortes-Ponce, C. Ferrer-Marin, T. Llaurado-Sabate, M. Llaurado-Vernet, P. Llobet-Azpitarte, C. Mangrane-Guillen, G. Muñoz-Alvarez, M. A. Oliver-Esteve, F. París-Palleja, M. Ricart-Sancho, E. Rivera-Manrique, A. Salva-Brusel, M. Soler-Pont, M. Timon-Torres, M. Boldu-Ortega GarciaTalarn, C. Elizalde-Rio, C. GarciaTalarn, M. Gorgues-Espasa, A. Guell-Coll, C. Hernandez-Nuñez, M. A. Naranjo-Orihuela, A. Odena-Estrade, I. Palou-Vall, L. Panades-Baldrich, A. Ribe-Miro, Y. Ortega-Vila, I. Pascual-Palacios, X. Perez-Cuit, M. Rodrigo-Gotor, M. Aliseda-Tienza, C. Anguera-Perpiña, J. Boj-Casajuana, J. J. Cabre-Vila, C. Chancho-Rodriguez, S. Dalmau-Vidal, M. Español-Pons, M. Garcia-Barco, R. Gonzalez-Perez, M. Huguet-Jacob, A. Isach-Subirana, E. Marti-Suau, M. Munte-Bigorra, M. Nolla-Mallafre, J. Pardo-Andujar, R. Pedret-Llaberia, L. Peralta-Encinas, P. Preixens-Vallinoto, E. Ras-Vidal, C. Rubio-Gascon, A. Reche-Martinez, R. Sagarra-Alamo, M. Sanchez-Marin, M. I. Sanchez-Oro, A. R. Silva-Orjuela, J. Vizcaino-Marin, L. Auge-Garcia, A. I. Castelao-Alvarez, F. Calamote-Manso, L. Clotas-Sancho, J. Ferre-Gras, M. Gasull-Gomis, A. Gonzalez-Garces, S. Gonzalez-Gonzalo, A. Llano-Sanchez, E. Nevot-Bueno, E. Ruiz-Morcillo, M. S. del Amo-Benito, E. Guijarro-Tapia, A. J. Lopez-Barea, A. Manresa-Font, I. Marsà-Gimènez, R. Moreno-Ramon, N. Nievas-Diaz, M. Prieto-Cid, M. Pujol-Porquera, C. Toledo-Peinado, M. L. Barrera-Uriarte, E. J. Borreguero-Guerrero, M. P. Castell-Montrull, R. E. Catalin, P. CentellesVelilla, J. Ferre-Rey, M. J. Forcadell-Peris, C. M. Fuentes-Bellido, O. Garcia-Gimeno, R. Landin-Delgado, J. Ledo-Garcia, E. Satue-Gracia, O. Briones-Carcedo, C. Rambla-Vidal, J. Robuste-Ingles, O. E. Arago-Albalate, V. R. Arnau-Adan, E. N. Balcells-Gonzalez, A. Boada-Tous, G. Cando-Guasch, J. Casajuana-Brunet, J. Castellvi-Baldira, R. Girona-Real, M. C. Grau-Perez, M. Medina-Clemente, A. Moreno-Lagunas, M. C. Moreno-Ortiz, E. Pay-Caro, A. M. Prats-Caellas, R. Profitos-Amiell, F. Rodriguez-Alonso, F. J. Sobrino-Perez, P. Sole-Barreras, M. L. Villaroya-Bullido

**Affiliations:** 1 Unitat de Suport a la Recerca Tarragona-Reus, Institut Universitari d’Investigació en Atenció Primària Jordi Gol (IDIAP Jordi Gol), Reus, Spain; 2 Study Group on Respiratory Tract Diseases (GEPAR), Institut Universitari d’Investigació en Atenció Primària Jordi Gol (IDIAP Jordi Gol), Barcelona, Spain; 3 School of Medicine and Health Sciences, Universitat Rovira i Virgili, Tarragona, Spain; 4 CAP Sant Pere-Institut Català de la Salut, C/Cami de Riudoms, 53-55, 43203 Reus, Tarragona, Spain; 5 CIBERobn Physiopathology of Obesity and Nutrition, Institute of Health Carlos III (ISCIII), Madrid, Spain; 6 NFOC group School of Medicine and Health Sciences, Universitat Rovira i Virgili, Tarragona, Spain

**Keywords:** Lung function test, Dietary patterns, Tobacco smoke

## Abstract

**Background:**

Diet can help preserve lung function in smokers, in addition to avoidance of smoking. The study aimed to evaluate associations between dietary patterns and lung function in smokers without respiratory disease.

**Methods:**

This cross-sectional study analysed baseline data from randomised representative smokers without respiratory disease (*n* = 207, aged 35–70 years), selected from 20 primary health-care centres. Participants completed a validated semi-quantitative food-frequency questionnaire. Dietary patterns were identified by Principal Component Analysis (PCA). Impaired lung function was defined as FVC <80% and/or FEV1 < 80% of predicted value and/or FEV1/FVC <0.7. Associations were determined by logistic regression.

**Results:**

Three major dietary patterns were identified. In multivariate-adjusted model, impaired lung function was associated with the Alcohol-consumption pattern (OR 4.56, 95% CI 1.58–13.18), especially in women (OR 11.47, 95% CI 2.25–58.47), and with the Westernised pattern in women (OR 5.62, 95% CI 1.17–27.02), whereas it not was associated with the Mediterranean-like pattern (OR 0.71, 95% CI 0.28–1.79).

**Conclusion:**

In smokers without respiratory disease, the Alcohol-consumption pattern and the Westernised pattern are associated with impaired lung function, especially in women. The Mediterranean-like pattern appears to be associated with preserved lung function because no statistical association is observed with impaired lung function. In addition to smoking cessation, modifying dietary patterns has possible clinical application to preserve lung function.

**Electronic supplementary material:**

The online version of this article (doi:10.1186/s12890-016-0326-x) contains supplementary material, which is available to authorized users.

## Background

Diet and nutrition are increasingly recognised as modifiable contributors to the prevention, development and progression of chronic diseases such as cancer and cardiovascular diseases [1, 2], but dietary impact on lung function is not well established. Observational and experimental studies have attempted to clarify the role of diet in maintaining lung function and reducing the risk of diseases such as chronic obstructive pulmonary disease (COPD) and asthma [3]. Smoking is the main cause of impaired lung function, although environmental agents, respiratory infections, genetic disorders, and eating habits may also be involved [4].

A protective action of certain foods and nutrients on various lung parameters has been described [5]. Fruit and vegetables intake is highly associated with respiratory health due to the benefits of antioxidant vitamins (C, D, E, and ß-carotene), minerals (magnesium, calcium, selenium, and potassium), dietary fibre and phytochemicals [6–9]. Omega-3 fatty acids, primarily eicosapentaenoic acid (C20:5) and docosahexaenoic acid (C22:6), found in oily fish and seafood have been shown to interfere with the body’s inflammatory response and may ward off some of the inflammatory mechanisms involved in the physiopathology of COPD, asthma, and obstructive lung disease [10, 11]. In contrast, high consumption of processed meat (bacon, gammon, ham, corned beef, spam and luncheon meat, sausage, and meat pies) is associated with worse lung function and with increased risk of COPD [12, 13]. Low-to-moderate alcohol consumption has been associated with improved lung function [14, 15], whereas excessive intake has detrimental effects [16], although the specific threshold remains undefined.

Although analysing the effect of individual foods has been valuable, there are conceptual and methodological limitations because actual meals consist of nutrients that are likely to interact or be synergistic [17]. Dietary pattern analysis addresses this issue by factor analysis of models containing interrelated variables (foods) as manifestations of composite factors that represent eating patterns in a study population and classify individuals according to the combination of foods they choose to consume [18]. Thus, dietary patterns provide a framework to examine the health effects of the whole diet. Research has shown that a “western-unhealthy diet” characterized by high intake of refined grains, cured and red meats, desserts/sweets, and French fries is positively associated with increased COPD risk, even after adjusting for age and total energy intake [19, 20]. On the other hand, a “prudent-healthy diet” rich in vegetables, fruits, fish, and whole grains is associated with better lung function, especially in men who smoke [21]. Thus, our hypothesis was that, in addition to the avoidance of smoking, a healthy dietary pattern could preserve lung function in smokers.

The aim of the present study was to identify major dietary patterns in a Spanish population of adult smokers without respiratory disease and to examine the association between dietary patterns and impaired lung function.

## Methods

### Study design

A cross-sectional study was performed in a sample of randomised representative smokers without respiratory disease with baseline data from the RESET study. RESET is a multicentre randomized controlled clinical trial carried out in 20 primary health care centres in the province of Tarragona (Spain), aimed to evaluate the 12-month effectiveness of an intervention providing structured information about the results of spirometry in prolonging smoking abstinence rates [22]. This trial has been registered with ClinicalTrial.gov (NCT02153047).

### Subjects

Inclusion criteria were 35 to 70 years of age, without respiratory symptoms, current smoker with a cumulative consumption of more than 10 pack-years, and signed informed consent. Exclusion criteria were any evidence of previous clinical diagnosis of respiratory disease or any chronic or terminal condition that would affect the baseline parameters or complicate the testing and analysis to be conducted during the study period.

### Assessment of variables

Baseline examination included a structured questionnaire designed to collect sociodemographic data, socioeconomic status, history of diseases and medications, symptoms, alcohol consumption (standard units/week), smoking status in cumulative pack-years (defined as the daily average of cigarettes smoked, multiplied by the number of years of smoking, divided by 20 cigarettes in a pack) and physical activity (sedentary, moderate or intense, according to frequency, minutes/week, type and amount of work-related physical activity).

Height (cm) and weight (to nearest 0.05 kg) were measured with the participant in light clothing and no shoes, using calibrated scales and a wall-mounted stadiometer, respectively. BMI was calculated as kg/m^2^. WC was measured midway between the lowest rib and the iliac crest using an anthropometric tape.

Dietary intake information was collected using a 45-item food-frequency questionnaire (FFQ) validated for the Spanish population [23]. Participants indicated their average frequency of consumption of food items for during the previous 12 months using specified categories for number of times/week and times/month.

### Definition of main outcome

The primary outcome was lung function status defined by impaired lung function. Lung function was evaluated by spirometry tests measuring forced vital capacity (FVC) and forced expiratory volume in one second (FEV1), using an ultrasound pneumotacograph (Datospir-600, SIBELMED, S.A.) and standardized procedure [24]. Impaired lung function was defined as FVC <80% of the predicted value and/or FEV1 < 80% of the predicted value and/or FEV1/FVC <0.7 [25].

### Sample size

The sample size requirements for this study were intended to provide adequate power for the analysis of the main outcome. Accepting an alpha risk of 0.05 in a two-sided test with 69 subjects in each group, the statistical power was 89 to recognize as statistically significant in the Alcohol-consumption pattern the difference between 10.1% (prevalence of impaired lung function in the lowest tertile) to 31.8% (prevalence of impaired lung function in the highest tertile) [GRANMO v7.12, IMIM software].

### Dietary patterns and statistical analysis

The 45-item FFQ was categorized into 19 food groups based on the similarity of nutrients (Additional file 1: Table S1) and dietary patterns were obtained using Principal Component Analysis (PCA) [23]. The Kaiser-Meyer-Olkin (KMO) and Bartlett test were used to test the adequacy of using PCA. We considered an Eigen value cut point of 1.5 as indicating a major dietary pattern. Varimax rotation was used to test the correlations between variables and factors. Finally, each dietary pattern was named according to the nature of the food groups included, as in previously published studies [26, 27].

The factor score for the dietary patterns was categorized into tertiles and the prevalence of impaired lung function was calculated for each tertile. Differences between dietary patterns groups were compared using chi-squared for qualitative variables and one-way analysis of variance (ANOVA) with post-hoc Bonferroni test for quantitative variables. We performed multivariate logistic regression to determine the association between dietary patterns and impaired lung function, obtaining the odds ratio (OR) and 95% confidence interval (CI) for the highest tertile of dietary pattern, compared with the lowest tertile. All logistic regression models were adjusted for the following confounding factors: age, sex, socioeconomic status, height, weight, waist circumference, physical activity, and cumulative tobacco use. Analyses were stratified by sex, testing for interactions of dietary pattern with sex and with smoking. Statistical tests were 2-sided at the 5% significance level. All statistical analyses were performed using SPSS software (version 22.0; SPSS Inc, Chicago).

## Results

Total study population was 207 participants (mean age 50.7 ± 9.0 years, 44.0% men). Cumulative tobacco consumption was 27.1 ± 16.3 pack-years. Spirometry showed marked change in pulmonary function parameters in 47 (22.7%) subjects, mostly mild in severity (72.3%): 17 (36.2%) obstructive, 14 (29.8%) restrictive and 16 (34.0%) of mixed type. Impaired lung function was more prevalent in men than in women (30.8% vs 16.4%; *p* = 0.014).

### Dietary patterns

The PCA identified three major dietary patterns that explained 31% of variation: Alcohol-consumption, Westernised, and Mediterranean-like pattern. The Alcohol-consumption pattern was loaded by intake of wine, beer, and/or distilled drinks (whisky, gin, cognac, etc.). The Westernised pattern was heavily loaded by high consumption of cured and red meats, dairy products, and sugary drinks, desserts and sweets, and low in fruits, vegetables, legumes, and fish. The Mediterranean-like pattern was heavily loaded by high intake of poultry, eggs, fish, vegetables, legumes, potatoes, dairy desserts, fruits, nuts, and dried fruit. Loading factors of food groups across these major patterns are presented in Table 1.

**Table 1:** Factor-loading matrix for 3 major dietary patterns among smokers without respiratory disease^1^

	Dietary patterns		
Food groups	Alcohol-consumption	Westernised	Mediterranean-like
Legumes	–	−0.29	0.52
Potatoes	–	–	0.49
Fish	–	−0.32	0.45
Eggs	0.21	–	0.44
Vegetables	–	−0.62	0.43
Dairy desserts	−0.22	–	0.48
Fruit	–	−0.55	0.41
Nuts and dried fruit	0.39	–	0.32
Poultry	−0.36	–	0.24
Refined grains	−0.30	0.38	0.46
Whole grains	–	0.41	0.41
Sweets and desserts	–	0.33	0.39
Cured and red meats	0.22	0.43	0.35
Sugary drinks	–	0.46	0.22
Dairy products	–	0.27	–
Low-calorie drinks	−0.25	–	–
Wine	0.63	–	–
Beer	0.59	–	–
Distilled drinks	0.42	–	−0.21
Percentage of variance explained (%)	8.3	9.9	12.8

^1^Values <0.20 were excluded for simplicity

Table 2 shows nutrient intake by tertile distribution of dietary patterns. Comparing the highest to the medium and lowest tertiles, the Alcohol-consumption pattern showed nearly twice the alcohol intake; Westernised pattern showed higher energy intake and carbohydrate, protein and fat (saturated and monounsaturated fatty acid) and cholesterol, and lower vitamin D; and Mediterranean-like pattern showed higher energy intake and carbohydrate, protein, fat (saturated, polyunsaturated and monounsaturated fatty acid), cholesterol, fibre, magnesium, calcium, iron, carotene and vitamins (retinol, folate, B-complex, C, D and E).

**Table 2:** Daily nutrient intake of all subjects by tertiles of dietary patterns

Nutrient intake	Alcohol-consumption pattern			Westernised pattern			Mediterranean-like pattern		
	Tertile 1	Tertile 2	Tertile 3	Tertile 1	Tertile 2	Tertile 3	Tertile 1	Tertile 2	Tertile 3
Energy intake, Kcal	2410.9 ± 319.2	2480.6 ± 296.1	2466.5 ± 269.7	2369.6 ± 267.2	2420.4 ± 276.8	2568.0 ± 308.9^#,¥^	2267.8 ± 215.1	2409.7 ± 237.6*	2680.7 ± 270.2^#,¥^
Carbohydrate, g	267.7 ± 58.3	268.3 ± 54.4	254.3 ± 42.7	251.7 ± 46.3	259.0 ± 50.5	279.6 ± 56.4^#,¥^	239.6 ± 41.0	258.3 ± 48.1	292.5 ± 53.4^#,¥^
Protein, g	83.1 ± 11.2	87.4 ± 10.6	86.5 ± 11.8	84.1 ± 11,6	84.2 ± 10.3	88.7 ± 11.7^#,¥^	77.0 ± 7.7	84.2 ± 7.5*	95.8 ± 9.7^#,¥^
Fat, g	107.7 ± 8.1	112.2 ± 8.4*	113.3 ± 9.9^#^	108.1 ± 8.7	109.8 ± 8.4	115.4 ± 8.7^#,¥^	104.5 ± 5.7	109.9 ± 6.1*	119.0 ± 8.7^#,¥^
SF, mg	30.8 ± 3.6	32.7 ± 3.7*	32.6 ± 4.1^#^	30.5 ± 3.7	31.6 ± 3.4	34.2 ± 3.6^#,¥^	29.7 ± 2.6	31.6 ± 3.2*	35.0 ± 3.8^#,¥^
MUFA, mg	56.8 ± 3.1	58.6 ± 3.2*	59.4 ± 3.9^#^	56.9 ± 3.3	57.8 ± 3.3	60.0 ± 3.4^#,¥^	55.7 ± 2.2	57.8 ± 2.3*	61.2 ± 3.4^#,¥^
PUFA, mg	11.8 ± 1.0	12.3 ± 1.1	12.7 ± 1.4^#^	12.2 ± 1.2	12.1 ± 1.2	12.5 ± 1.2	11.4 ± 0.7	12.1 ± 0.7*	13.3 ± 1.1^#,¥^
Cholesterol, mg	323.6 ± 61.2	356.2 ± 54.8*	365.3 ± 78.8^#^	332.3 ± 66.1	337.9 ± 67.9	374.8 ± 62.2^#,¥^	313.3 ± 48.7	334.1 ± 48.6	397.8 ± 72.6^#,¥^
Fibre, g	18.8 ± 3.5	18.7 ± 3.5	18.6 ± 3.2	19.4 ± 3.4	18.3 ± 3.4	18.4 ± 3.5	16.4 ± 2.6	18.5 ± 2.8*	21.2 ± 3.0^#,¥^
Alcohol, g	5.51 ± 3.8	6.83 ± 4.0	11.93 ± 6.3^#,¥^	7.46 ± 5.1	8.55 ± 5.5	8.26 ± 6.1	8.95 ± 5.6	7.23 ± 5.1	8.09 ± 5.9
Mg, mg	277.6 ± 40.9	286.6 ± 41.5	299.3 ± 43.2^#^	285.9 ± 44.8	280.6 ± 40.5	297.0 ± 41.3	256,2 ± 29.3	283.0 ± 28.8*	324.4 ± 37.7^#,¥^
Ca, mg	848.1 ± 198.3	863.8 ± 175.5	938.1 ± 170.1^#^	867.8 ± 208.3	866.5 ± 178.3	915.7 ± 164.1	806.5 ± 165.8	873.7 ± 179.4	969.7 ± 174.3^#,¥^
Fe, mg	11.8 ± 1.9	12.0 ± 1.7	12.4 ± 1.7	11.8 ± 1.8	11.8 ± 1.7*	12.5 ± 1.8^#,¥^	10.6 ± 1.1	11.8 ± 1.1*	13.7 ± 1.5^#,¥^
β-carotene, mg	2241.1 ± 507.3	2210.7 ± 584.9	2178.3 ± 539.9	2549.2 ± 522.8	2162.5 ± 488.7	1918.3 ± 418.8^#,¥^	1879.2 ± 431.8	2270.2 ± 499.1*	2480.7 ± 518.0^#,¥^
Retinol, mcg	504.2 ± 111.9	559.1 ± 106.5*	552.4 ± 107.6^#^	497.6 ± 91.6	527.7 ± 102.3	590.4 ± 117.8^#,¥^	493.5 ± 69.7	527.0 ± 106.4	595.3 ± 125.0^#,¥^
Folate, ng	289.7 ± 47.4	296.1 ± 51.3	302.6 ± 51.6	314.9 ± 54.4	290.3 ± 47.2*	283.2 ± 43.3^#^	260.3 ± 35.4	291.4 ± 33.5*	336.7 ± 47.4*
Vitamin B1, mg	1.32 ± 0.2	1.39 ± 0.2	1.42 ± 0.2^#^	1.34 ± 0.2	1.37 ± 0.2	1.41 ± 0.2	1.24 ± 0.2	1.36 ± 0.1*	1.54 ± 0.2^#,¥^
Vitamin B6, mg	1.90 ± 0.3	1.94 ± 0.3	1.99 ± 0.3	1.97 ± 0.3	1.91 ± 0.3	1.95 ± 0.3	1.73 ± 0.2	1.91 ± 0.2*	2.18 ± 0.2^#,¥^
Vitamin B12, mg	6.58 ± 1.1	7.04 ± 1.1*	7.03 ± 1.1	6.68 ± 1.1	6.72 ± 1.0	7.25 ± 1.2^#,¥^	6.29 ± 0.8	6.74 ± 0.9*	7.63 ± 1.2^#,¥^
Vitamin C, mg	98.1 ± 25.5	97.9 ± 30.6	97.2 ± 26.2	114.4 ± 26.5	92.6 ± 26.5*	86.1 ± 20.6^#^	81.3 ± 22.0	97.8 ± 24.8*	114.1 ± 25.0^#,¥^
Vitamin D, mcg	2.96 ± 0.7	2.87 ± 0.7	2.73 ± 0.7	3.19 ± 0.7	2.71 ± 0.6*	2.66 ± 0.7^#,¥^	2.60 ± 0.6	2.72 ± 0.5	3.25 ± 0.8^#,¥^
Vitamin E, mg	11.0 ± 0.8	11.3 ± 1.0	11.6 ± 1.2^#^	11.5 ± 1.1	11.1 ± 1.0*	11.2 ± 1.0	10.5 ± 0.6	11.2 ± 0.7*	12.1 ± 1.0^#,¥^

Data are presented as median ± standard deviation (SD)

*Abbreviations*: *SF* saturated fatty acid, *MUFA* monounsaturated fatty acid, *PUFA* polyunsaturated fatty acid, *Mg* magnesium, *Ca* calcium, *Fe* iron

(*) *p*-value ≤ 0.05. Analysis of variance has been performed, and compares tertile 1 (lowest) and tertile 2 (medium)

(^#^) *p*-value ≤ 0.05. Analysis of variance has been performed, and compares tertile 1 (lowest) and tertile 3 (highest)

(^¥^) *p*-value ≤ 0.05. Analysis of variance has been performed, and compares tertile 2 (medium) and tertile 3 (highest

### Participant characteristics across tertiles of each dietary pattern

Characteristics of participants according to tertiles of dietary patterns are shown in Table 3. We detected interaction between sex and Westernised pattern (*p* = 0.011), for this reason analyses were stratified by sex. In the Alcohol-consumption pattern, women had lower weight and BMI values in the highest *vs* the lowest tertile (ANOVA, *p* = 0.024 and *p* = 0.015; respectively). In the Westernised pattern, overall population in highest tertile were younger, and presented lower BMI and waist circumference values compared to the lowest tertile (ANOVA, *p* < 0.001, *p* = 0.009 and *p* = 0.045; respectively). In the Mediterranean-like pattern, women in highest tertile had lower weight and BMI values compared with the intermediate tertile (ANOVA, *p* = 0.035 and *p* = 0.029; respectively).

**Table 3:** General characteristics of subjects according to tertiles of major dietary patterns

	Alcohol-consumption pattern			Westernised pattern			Mediterranean-like pattern		
	Tertile 1	Tertile 2	Tertile 3	Tertile 1	Tertile 2	Tertile 3	Tertile 1	Tertile 2	Tertile 3
All, *n* = 207									
Subjects (%)	69 (33.3)	69 (33.3)	69 (33.3)	69 (33.3)	69 (33.3)	69 (33.3)	69 (33.3)	69 (33.3)	69 (33.3)
Age (year)	49.7 ± 8.4	50.7 ± 8.6	51.8 ± 9.9	53.4 ± 8.6	52.2 ± 8.1	46.6 ± 8.8^#,¥^	52.5 ± 9.0	50.5 ± 8.2	49.1 ± 9.5
Anthropometric measures									
Weight (kg) Weight (kg)	75.6 ± 15.7	73.9 ± 18.5	74.3 ± 15.0	76.5 ± 15.9	74.5 ± 16.1	72.9 ± 17.2	74.2 ± 16.2	77.4 ± 16.0	72.2 ± 17.0
Height (cm)	162.3 ± 9.2	163.9 ± 9.9	166.5 ± 9.0^#^	162.4 ± 9.2	164.0 ± 9.7	166.2 ± 9.3	163.1 ± 9.8	165.0 ± 10.0	164.6 ± 8.6
BMI (kg/m2)	28.7 ± 5.2	27.4 ± 5.7	26.7 ± 4.5	28.9 ± 5.1	27.6 ± 4.8	26.3 ± 5.4^#^	27.8 ± 4.9	28.4 ± 5.3	26.5 ± 5.2
Waist circumference (cm)	96.8 ± 13.8	93.7 ± 14.1	94.7 ± 12.1	97.4 ± 12.8	95.9 ± 13.2	91.9 ± 13.7^#^	96.3 ± 12.5	96.6 ± 14.3	92.4 ± 13.0
Socioeconomic status									
Low	24 (34.8)	28 (40.5)	21 (30.4)	25 (36.2)	24 (34.7)	24 (34.7)	24 (34.7)	22 (31.8)	27 (39.1)
Medium	32 (46.4)	27 (39.1)	25 (36.2)	32 (46.3)	29 (42.0)	23 (33.3)	24 (34.8)	35 (50.7)	25 (36.2)
High	3 (4.3)	8 (11.6)	16 (23.2)	9 (13.0)	8 (11.6)	10 (14.5)	14 (20.3)	5 (7.2)	8 (11.6)
Physical Activity									
Sedentary	36 (52.2)	32 (46.4)	35 (50.7)	36 (52.2)	34 (49.3)	33 (47.8)	42 (60.9)	36 (52.2)	25 (36.2)
Moderate	27 (39.1)	33 (47.8)	28 (40.6)	26 (37.7)	33 (47.8)	29 (42.0)	26 (37.7)	24 (34.8)	38 (55.1)
Intense	6 (8.7)	4 (5.8)	6 (8.7)	7 (10.1)	2 (2.9)	7 (10.1)	1 (1.4)	9 (13.0)	6 (8.7)
Smoking									
Cumulative consumption (packets-year)	27.8 ± 17.1	25.4 ± 13.4	28.0 ± 18.0	26.9 ± 16.9	29.4 ± 15.7	24.8 ± 16.0	29.0 ± 15.3	28.1 ± 15.9	24.1 ± 17.3
Lung function parameters									
FVC, % of predicted	94.8 ± 14.9	94.3 ± 19.2	89.6 ± 17.0	92.4 ± 17.2	91.2 ± 15.7	95.1 ± 18.6	92.2 ± 17.8	93.6 ± 18.2	92.9 ± 15.6
FEV1, % of predicted	98.9 ± 14.7	97.3 ± 19.2	93.1 ± 19.2	96.9 ± 18.3	95.7 ± 17.4	96.8 ± 18.3	95.7 ± 19.3	96.6 ± 18.5	97.0 ± 16.0
FEV1/FVC ratio (%)	80.1 ± 6.6	78.8 ± 6.1	78.7 ± 8.0	79.4 ± 6.9	79.5 ± 6.7	78.7 ± 7.3	78.7 ± 6.7	78.7 ± 6.6	80.3 ± 7.5
Women, *n* = 116									
Subjects (%)	49 (42.2)	40 (34.5)	27 (23.3)	45 (38.8)	38 (32.8)	33 (28.4)	41 (35.3)	37 (31.9)	38 (32.8)
Age (year)	49.1 ± 9.0	50.4 ± 7.9	49.9 ± 9.1	51.5 ± 8.4	51.5 ± 8.1	45.2 ± 7.8^#,¥^	51.0 ± 9.1	51.2 ± 6.6	46.9 ± 9.1
Anthropometric measures									
Weight (kg)	71.6 ± 12.9	65.2 ± 14.2	63.1 ± 11.8^#^	70.4 ± 14.6	67.4 ± 13.3	63.5 ± 11.6	68.6 ± 14.0	70.8 ± 15.1	62.9 ± 10.2^,¥^
Height (cm)	158.3 ± 7.1	158.5 ± 7.9	158.6 ± 4.6	158.1 ± 7.9	158.3 ± 7.0	159.1 ± 5.2	157.6 ± 7.1	159.0 ± 7.7	158.9 ± 5.8
BMI (kg/m2)	28.6 ± 5.0	26.1 ± 5.8	25.1 ± 4.5^#^	28.2 ± 5.5	27.0 ± 5.4	25.2 ± 4.9^#^	27.6 ± 5.2	28.2 ± 6.2	25.0 ± 4.2^,¥^
Waist circumference (cm)	95.4 ± 13.4	89.8 ± 13.8	88.4 ± 11.7	94.2 ± 13.4	93.2 ± 13.3	87.0 ± 12.9	93.6 ± 13.1	93.8 ± 14.9	88.0 ± 11.7
Socioeconomic status									
Low	16 (32.6)	15 (37.5)	11 (40.7)	17 (37.7)	13 (34.3)	12 (36.4)	14 (34.2)	11 (29.7)	17 (44.7)
Medium	24 (49.0)	16 (40)	6 (22.2)	20 (44.4)	17 (44.8)	9 (27.3)	16 (39)	17 (45.9)	16 (39)
High	2 (4.0)	5 (12.5)	5 (18.5)	6 (13.3)	2 (5.3)	4 (12.1)	7 (17.1)	2 (5.4)	7 (17.1)
Physical Activity									
Sedentary	29 (59.2)	22 (55.0)	17 (63.0)	26 (57.8)	24 (63.2)	18 (54.5)	27 (65.9)	28 (75.7)	27 (65.9)
Moderate	18 (36.7)	16 (40.0)	9 (33.3)	16 (35.6)	14 (36.8)	13 (39.4)	13 (31.7)	8 (21.6)	13 (31.7)
Intense	2 (4.1)	2 (5.0)	1 (3.7)	3 (6.7)	0 (0.0)	2 (6.1)	1 (2.4)	1 (2.7)	1 (2.4)
Smoking									
Cumulative consumption (packets-year)	24.0 ± 14.6	23.8 ± 11.1	22.8 ± 15.3	24.0 ± 15.0	25.8 ± 12.8	20.8 ± 12.1	24.5 ± 11.9	24.8 ± 12.7	21.6 ± 15.8
Lung function parameters									
FVC, %	95.9 ± 15.4	98.9 ± 20.6	93.3 ± 16.6	97.8 ± 16.4	94.1 ± 14.9	96.9 ± 21.9	96.4 ± 17.9	97.8 ± 17.8	94.8 ± 17.4
FEV1, %	100.1 ± 15.4	100.3 ± 20.7	96.8 ± 22.1	102.3 ± 17.4	98.0 ± 17.9	97.0 ± 21.7	99.8 ± 20.1	99.2 ± 18.9	99.2 ± 17.9
FEV1/FVC ratio (%)	80.7 ± 7.3	78.1 ± 7.0	79.3 ± 9.6	80.1 ± 7.7	79.4 ± 7.6	78.6 ± 8.4	79.2 ± 7.7	77.6 ± 7.4	81.5 ± 8.1
Men, *n* = 91									
Subjects (%)	20 (22.0)	29 (31.9)	42 (46.2)	24 (26.4)	31 (34.1)	36 (39.6)	28 (30.8)	32 (35.2)	31 (34.1)
Age (year)	51.2 ± 6.7	51.1 ± 9.7	53.1 ± 10.4	56.9 ± 8.0	53.0 ± 8.2	47.9 ± 9.6^#^	54.9 ± 8.5	49.6 ± 9.8	51.9 ± 9.4
Anthropometric measures									
Weight (kg)	85.4 ± 17.9	85.8 ± 17.2	81.5 ± 12.2	87.9 ± 11.7	83.2 ± 15.0	81.5 ± 17.2	82.5 ± 15.8	85.0 ± 13.5	83.6 ± 16.6
Height (cm)	172.0 ± 6.0	171.2 ± 7.3	171.5 ± 7.3	170.5 ± 4.9	171.0 ± 7.9	172.7 ± 7.3	171.1 ± 7.4	171.9 ± 7.6	171.5 ± 6.1
BMI (kg/m2)	28.8 ± 5.6	29.2 ± 5.1	27.8 ± 4.2	30.3 ± 3.8	28.3 ± 4.0	27.3 ± 5.7	28.1 ± 4.5	28.8 ± 4.2	28.4 ± 5.7
Waist circumference (cm)	100.4 ± 14.6	99.1 ± 12.8	98.8 ± 10.6	103.4 ± 9.1	99.3 ± 12.5	96.4 ± 13.0	100.1 ± 10.6	99.9 ± 13.0	97.7 ± 12.7
Socioeconomic status									
Low	8 (40.0)	13 (44.8)	10 (23.8)	8 (33.4)	11 (35.5)	13 (33.3)	10 (35.7)	11 (34.7)	10 (32.3)
Medium	8 (40.0)	11 (37.9)	19 (45.2)	12 (50.0)	12 (38.7)	14 (38.9)	8 (28.6)	18 (56.2)	12 (38.8)
High	1 (5.0)	3 (10.3)	11 (26.2)	3 (12.5)	6 (19.4)	6 (16.7)	7 (25)	3 (9.4)	5 (16.2)
Physical Activity									
Sedentary	7 (35.0)	10 (34.5)	18 (42.9)	10 (41.7)	10 (32.3)	15 (41.7)	15 (53.6)	8 (25.0)	12 (38.7)
Moderate	9 (45.0)	17 (58.6)	19 (45.2)	10 (41.7)	19 (61.3)	16 (44.4)	13 (46.4)	16 (50.0)	16 (51.6)
Intense	4 (20.0)	2 (6.9)	5 (11.9)	4 (16.7)	2 (6.5)	5 (13.9)	0 (0.0)	8 (25.0)	3 (9.7)
Smoking									
Cumulative consumption (packets-year)	37.1 ± 19.6	27.6 ± 16.0	31.3 ± 19.0	32.4 ± 19.2	33.9 ± 17.9	28.5 ± 18.4	35.6 ± 17.4	31.9 ± 18.5	27.1 ± 18.7
Lung function parameters									
FVC, % of predicted	92.1 ± 13.3	87.9 ± 15.4	87.2 ± 17.0	82.3 ± 14.1	87.6 ± 16.1	93.4 ± 15.2^#^	86.0 ± 16.1	88.8 ± 17.7	90.5 ± 13.1
FEV1, % of predicted	96.1 ± 12.7	93.2 ± 16.5	90.7 ± 17.0	86.7 ± 15.6	92.8 ± 16.5	96.5 ± 14.9	89.7 ± 16.7	93.6 ± 17.9	94.4 ± 13.0
FEV1/FVC ratio (%)	78.8 ± 4.3	79.9 ± 4.5	78.3 ± 6.9	78.1 ± 5.2	79.6 ± 5.5	78.8 ± 6.3	77.9 ± 4.8	80.0 ± 5.4	78.7 ± 6.7

Data are presented as n (%) or mean ± standard deviation (DS) or median ± range depending on the type of variable

*Abbreviations*: *BMI* body mass index, *FVC* forced vital capacity % of predicted, *FEV1* maximum expiratory volume in the first second of a forced exhalation % of predicted

(*) *p*-value ≤ 0.05. Analysis of variance has been performed, and compares tertile 1 (lowest) and tertile 2 (medium)

(^#^) *p*-value ≤ 0.05. Analysis of variance has been performed, and compares tertile 1 (lowest) and tertile 3 (highest)

(^¥^) *p*-value ≤ 0.05. Analysis of variance has been performed, and compares tertile 2 (medium) and tertile 3 (highest)

### Dietary pattern and impaired lung function

Prevalence of impaired lung function across tertiles of dietary patterns is presented in Table 4. More than two or three times prevalence of impaired lung function in medium and highest tertiles of the Alcohol-consumption pattern, compared with the lowest (31.8 and 26.0 vs. 10.1, respectively; *p* = 0.007); the difference between tertiles was more intense in women than in men. Women in lowest and medium tertiles of the Westernised pattern had a lower prevalence of impaired lung function compared to the highest, but no significant differences. However, twice prevalence of men in the highest tertile of the Westernised pattern compared to the lowest and medium tertile had impaired lung function (54.1 vs. 22.6 and 22.2, respectively; *p* = 0.015). Without reaching significance, more subjects in the highest tertile of the Mediterranean-like pattern had preserved lung function compared to medium and lowest tertiles.

**Table 4:** Impaired lung function prevalence (and 95% confidence intervals) across tertiles (T1-T3) of major dietary patterns

	Terti1e 1		Terti1e 2		Terti1e 3		*p*-value
	%	95% CI	%	95% CI	%	95% CI	
All (*n* = 207)							
Alcohol-consumption pattern	10.1	5.0–19.5	26.0	17.2–37.5	31.8	22.1–43.6	0.007
Westernised pattern	26.0	17.2–37.5	18.8	11.4–29.6	23.2	14.8–34.4	0.593
Mediterranean-like pattern	27.5	18.4–39.0	23.2	14.8–34.4	17.4	10.2–28.0	0.361
Women (*n* = 116)							
Alcohol-consumption pattern	6.1	2.1–16.5	17.5	8.7–31.9	33.3	18.6–52.2	0.009
Westernised pattern	11.1	4.8–23.5	15.8	7.4–30.4	24.2	12.8–41.0	0.300
Mediterranean-like pattern	22.0	12.0–26.7	16.2	7.7–31.1	10.5	4.2–24.1	0. 391
Men (*n* = 91)							
Alcohol-consumption pattern	20.0	8.1–41.6	37.9	22.7–56.0	31.0	19.1–46.0	0. 409
Westernised pattern	54.1	35.1–72.1	22.6	11.4–39.8	22.2	11.7–38.1	0.015
Mediterranean-like pattern	35.7	20.7–54.2	31.2	18.0–48.6	25.8	13.7–43.2	0. 711

*p*-values are obtained by chi-squared test, and compared tertile 1 (lowest), tertile 2 (medium) and tertile 3 (highest)

The multivariate-adjusted model in Table 5 shows the odds for impaired lung function across tertiles of dietary patterns controlled by confounding factors including demographics covariates (age and sex), socioeconomic status, anthropometrics measurements (height, weight and waist circumference), physical activity, energy intake and smoking behaviour (accumulated consumption in pack-years). Alcohol-consumption pattern was associated with impaired lung function overall (tertile-3: OR 4.56, 95% CI 1.58–13.18; *p* = 0.005), but especially in women (tertile-3: OR 11.47, 95% CI 2.25–58.47; *p* = 0.003). The Westernised pattern had greater risk of impaired lung function only in women (tertile-3: OR 5.62, 95%CI 1.17–27.02; *p* = 0.031). The Mediterranean-like pattern was associated with a trend for preserved lung function (tertile-3: OR 0.71; 95%CI 0.28–1.79).

**Table 5:** Multivariate-adjusted odds ratios (95% confidence intervals) for impaired lung function across tertiles of major dietary patterns

	Impaired lung function					
	All^a^		Women^b^		Men^b^	
	OR (95% CI)	*p*-value	OR (95% CI)	*p*-value	OR (95% CI)	*p*-value
Alcohol-consumption pattern						
Tertile 1	1		1		1	
Tertile 2	4.17 (1.45–11.95)	0.008	4.43 (0.96–20.51)	0.057	4.50 (0.79–25.53)	0.090
Tertile 3	4.67 (1.61–13.57)	0.005	12.33 (2.34–64.96)	0.003	2.60 (0.50–13.43)	0.256
Westernised pattern						
Tertile 1	1		1		1	
Tertile 2	0.63 (0.25–1.56)	0.315	1.31 (0.33–5.31)	0.700	0.28 (0.07–1.10)	0.068
Tertile 3	1.42 (0.52–3.83)	0.494	6.33 (1.27–31.66)	0.025	0.49 (0.12–2.01)	0.321
Mediterranean-like pattern						
Tertile 1	1		1		1	
Tertile 2	0.91 (0.38–2.19)	0.838	0.52 (0.13–1.99)	0.340	1.07 (0.28–4.16)	0.916
Tertile 3	0.69 (0.23–2.04)	0.502	0.29 (0.05–1.78)	0.180	0.92 (0.21–4.01)	0.908

*Abbreviations*: *OR* odds ratio, *CI* confidence interval

*p*-values are obtained by multiple logistic regression, and compared tertile 1 (lowest), tertile 2 (medium) and tertile 3 (highest)

^a^Adjusted for sex, age, weight, height, waist circumference, cumulative consumption, social class, physical activity and energy intake

^b^Adjusted for age, weight, height, waist circumference, cumulative consumption, social class, physical activity and energy intake

## Discussion

Limited data are available on the associations between food patterns and lung function in adults, and more evidence is required from studies of clinical interventions to confirm their effectiveness in preventing respiratory disease [5]. In the present study, three major dietary patterns were derived in smokers without pulmonary disease: Alcohol-consumption pattern, Westernised pattern, and Mediterranean-like pattern. Impaired lung function was observed in all participants with an Alcohol-consumption pattern, but especially in women. The Westernised pattern was associated with impaired lung function in women. In contrast, the Mediterranean-like dietary pattern was observed with favourable effects because no statistical association was detected with impaired lung function.

Our Alcohol-consumption pattern was strongly associated with impaired lung function in the total sample, but especially in women. It is known that alcohol intake can have an impact on the incidence of diseases and other health conditions, such as respiratory health [5, 14], but the present study is the first that associates an alcohol dietary pattern with impaired lung function. Daily alcohol consumption was higher in the highest tertile subjects compared with the lowest tertile (11.7 ± 6.3 g/d vs 5.6 ± 3.8 g/d), although according to the WHO ‘International guide for monitoring alcohol consumption and related harm’ it was still less than half of the upper limit (<40 g/d for men and <20 g/d for women) [28]. Apart from environmental factors, the alcohol-related harm is determined by three related dimensions of drinking: the volume of alcohol consumed, the pattern of drinking, and the quality of the alcohol type consumed [29]. Brief exposure to recommended alcohol consumption can be healthy for lung function due to the antioxidant effect, especially red wine for its high polyphenol content (tannins) that may enhance mucociliary clearing, stimulate bronchodilation, and attenuate the airway inflammation and injury observed in asthma and COPD [15, 30]. However, prolonged and heavy exposure to alcohol impairs mucociliary clearing, may complicate asthma management, and likely worsens outcomes including lung function and mortality in COPD patients. The present study had two limitations in this regard. First, we had no data to assess whether the increased consumption of alcohol represented red wine, beer or spirits and could not establish a relationship between the type of beverage and lung function. Second, it is not possible determine if women who drank more alcohol share other unknown characteristics responsible for their impaired lung function.

The finding that our Westernised pattern had a negative impact on lung function is consistent with epidemiologic studies reporting a deleterious effect on lung function of a “western” diet [19, 20, 31, 32]. The deleterious effect of this pattern is based on the high intake of nitrites from meat and processed meat [12, 13]. Nitrites generate reactive nitrogen species in the respiratory tract that may cause damage, producing structural changes resembling emphysema, amplification of inflammatory processes in the airways, and lung parenchyma causing DNA damage, inhibition of mitochondrial respiration, protein dysfunction, and cell damage [33]. However, the role of dietary nitrites and nitrates in health is a matter of debate [34]. Nevertheless, in our cohort, in the multivariate model, the harmful effect of the Westernised pattern on lung function was only observed in women. Several reasons could explain this gender difference. First, although data from large epidemiological studies show that susceptibility to tobacco is similar in both sexes, some authors suggest that women may be biologically more vulnerable to the adverse effects of smoking than men, due to sex differences in metabolising cigarette smoke [35, 36]. Second, age could be a factor because deterioration of lung function in smokers is not immediate and most patients require years of consumption to observe changes in the spirometry results, thus older people are more susceptible than younger adults [35]. Men at the highest tertile of the Westernised pattern were younger than those in the lowest tertile; thus, age could have been a factor in the low prevalence of impaired lung function observed in the present study. Finally, this gender difference could be explained by the observed differences in daily vitamin D intake. Although all participants consumed less than the current recommendation of 15 ± 10 mcg per day [37], daily Vitamin D intake was lower in the highest tertile of the Westernised pattern overall, but especially in women (3.3 vs 2.7 mcg/d in the lowest and highest tertile, respectively; *p* = 0.037, data not shown); this difference was not observed in men (3.1 vs 2.5 mcg/d, respectively; *p* = 0.182, data not shown). Given the pleiotropic actions of vitamin D, it is biologically plausible that low consumption partially led to a serum deficit of this vitamin, which could play a pathogenic role in the development of various respiratory diseases or contribute to a worse prognosis [38, 39]. The mechanism by which serum vitamin D deficiency could impair lung function may be its protective immunomodulatory effects and/or its action on muscle [5]. The question remains whether low vitamin D levels contribute to the aetiology of lung disease or if vitamin D deficiency is simply a manifestation of the lung disease and/or its treatment [40]. As research in this field unfolds, vitamin D consumption or/and serum concentrations should be evaluated in larger trials focused on specific respiratory diseases.

The Mediterranean-like pattern was associated with a favourable effect on lung function, and no statistically significant difference was observed in the prevalence of impaired lung function. Nonetheless, these protective lung findings are in keeping with results of prior studies that found that a similar composition pattern named prudent pattern (high consumption of fruit, vegetables, oily fish, and wholemeal cereals) may protect against impaired lung function and COPD [21]. All data provided by present study suggest that a healthy diet may preserve in lung function against deleterious effects of a smoking habit, supported by no association between Mediterranean-like pattern and impaired lung function. The Mediterranean-like pattern in our study included fruits, vegetables, nuts, fish, and olive oil, all of which are part of the traditional Mediterranean Diet (MD) [41]; however, it also included consumption of red and processed meats, desserts, sweets, and refined grains. Probably this was the reason that the Mediterranean-like pattern, the healthiest pattern in our study, was not healthy enough to obtain significant reducing in impaired lung function prevalence in smokers with a high adherence to this pattern. To show the effect of this MD, intervention studies are needed in smokers without respiratory symptoms [42].

In addition to the stated limitations, the sample of this study also must be considered. The sample size possibly limited the statistical power to detect some differences between the patterns and motivated that some ORs confidence intervals obtained in the multivariate model were very large (for example: 2.34–64.96). However, the present study had several strengths. Our observational cross-sectional study is the first in the Spanish population to analyse the association of dietary patterns with lung function, providing new information that could lead to clinical application of dietary modification as a tool to preserve lung function in smokers without pulmonary disease, in addition to encouraging the avoidance of smoking [42]. Dietary patterns were derived using statistical techniques to summarize dietary exposure and PCA showed patterns in foods loaded and factor loadings similar to other patterns found in previous studies [5]. Using dietary patterns in nutritional epidemiology instead of analysing individual foods and nutrients has conceptual and methodological arguments in favour. Dietary patterns analysis takes account of interactions between nutrients, thus allowing consideration of the effect of the whole diet [18]. Using PCA, we were able to fit a model with low noise sensitivity, decreased requirements for capacity and memory, and increased efficiency given the processes taking place in a smaller dimension [26]. Finally, our study compared to previous studies is that we had objective measures of lung function with spirometry and post-bronchodilator tests that avoid potential problems of bias from self-reported characteristics [34].

## Conclusion

In smokers without respiratory disease, an Alcohol-consumption pattern is associated with impaired lung function overall population, a Westernised pattern reduced lung function in women, and a Mediterranean-like pattern appears to be associated with preserved lung function. Although preventive efforts must focus on smoking cessation, dietary patterns may also protect lung function.

## Additional file

Additional file 1: Table S1. Categorization of the 45-item FFQ into food groups (DOC 37 kb)

## Abbreviations

ANOVA: Analysis of variance
BMI: Body mass index
CI: Confidence interval
COPD: Chronic obstructive pulmonary diseases
FEV1: Forced expiratory volume in 1 s
FFQ: Food-frequency questionnaire
FVC: Forced vital capacity
MD: Mediterranean diet
MUFA: monounsaturated fatty acids
OR: Odds ratio
PCA: Principal component analysis
PUFA: Polyunsaturated fatty acids
WHO: World Health Organization
