# Supplementary material for: Dietary patterns are associated with lung function among Spanish smokers without respiratory disease
Source: BMC Pulm Med. 2016 Nov 25;16:162. doi: 10.1186/s12890-016-0326-x (PMC5123418; doi:10.1186/s12890-016-0326-x)
Supplement: Additional file 1: Table S1. — Categorization of the 45-item FFQ into food groups (DOC 37 kb) [file 12890_2016_326_MOESM1_ESM.doc]

Supplementary file 1. Categorization of the 45-item FFQ into food groups

| **Food groups** | **Food items** |
| --- | --- |
| Cured and red meats | Beef, pork, lamb, sausages, minced meat, hamburger, ham, salted ham |
| Poultry | Chicken and turkey with or without skin |
| Fish | Oily fish and other fish, seafood |
| Eggs | Eggs |
| Dairy products | Milk, yogurt, cheese |
| Dairy desserts | Custard, flan, ice cream |
| Fruit | Apple, pear, peaches, nectarine, bananas, watermelon and citrus fruit: oranges, tangerine, strawberries, kiwi.  Natural fruit juice |
| Nuts and dried fruit | Almonds, hazelnuts, walnuts, peanuts |
| Vegetables | Salad: lettuce, tomato, endive, carrots  Green beans, chard, spinach, garlic,  Garnish vegetables: eggplant, mushrooms |
| Legumes | Beans, peas, lentils, soy |
| Potatoes | Potatoes, snacks (French fries / chips) |
| Whole grains | White breads, pasta, rice, processed food |
| Refined grains | Breakfast cereals, cookies, muffins |
| Sweets and desserts | Chocolates, cakes, cream cakes, donuts, jams, candies, jelly beans |
| Sugary drinks | Soft drinks, commercial fruit juice |
| Low-calorie drinks | Low-calorie soft drinks, beer without alcohol |
| Wine | Wine, sangria |
| Beer | With alcohol |
| Drinks, distilled | Whisky, gin, cognac |
